# Framingham Heart Study genome-wide association: results for pulmonary function measures

**DOI:** 10.1186/1471-2350-8-S1-S8

**Published:** 2007-09-19

**Authors:** Jemma B Wilk, Robert E Walter, Jason M Laramie, Daniel J Gottlieb, George T O'Connor

**Affiliations:** 1 Department of Neurology, Boston University School of Medicine, Boston, MA, USA; 2 Pulmonary Center, Department of Medicine, Boston University School of Medicine, Boston, MA, USA; 3 The National Heart, Lung, and Blood Institute's Framingham Heart Study, Framingham, MA, USA; 4 Program in Bioinformatics, Boston University, Boston, MA, USA; 5 VA Boston Healthcare System, Boston, MA, USA

## Abstract

**Background:**

Pulmonary function measures obtained by spirometry are used to diagnose chronic obstructive pulmonary disease (COPD) and are highly heritable. We conducted genome-wide association (GWA) analyses (Affymetrix 100K SNP GeneChip) for measures of lung function in the Framingham Heart Study.

**Methods:**

Ten spirometry phenotypes including percent of predicted measures, mean spirometry measures over two examinations, and rates of change based on forced expiratory volume in one second (FEV_1_), forced vital capacity (FVC), forced expiratory flow from the 25^th ^to 75^th ^percentile (FEF_25–75_), the FEV_1_/FVC ratio, and the FEF_25–75_/FVC ratio were examined. Percent predicted phenotypes were created using each participant's latest exam with spirometry. Predicted lung function was estimated using models defined in the set of healthy never-smokers, and standardized residuals of percent predicted measures were created adjusting for smoking status, pack-years, and body mass index (BMI). All modeling was performed stratified by sex and cohort. Mean spirometry phenotypes were created using data from two examinations and adjusting for age, BMI, height, smoking and pack-years. Change in pulmonary function over time was studied using two to four examinations with spirometry to calculate slopes, which were then adjusted for age, height, smoking and pack-years.

**Results:**

Analyses were restricted to 70,987 autosomal SNPs with minor allele frequency ≥ 10%, genotype call rate ≥ 80%, and Hardy-Weinberg equilibrium p-value ≥ 0.001. A SNP in the interleukin 6 receptor (*IL6R*) on chromosome 1 was among the best results for percent predicted FEF_25–75_. A non-synonymous coding SNP in glutathione S-transferase omega 2 (*GSTO2*) on chromosome 10 had top-ranked results studying the mean FEV_1 _and FVC measurements from two examinations. SNPs nearby the *SOD3 *and vitamin D binding protein genes, candidate genes for COPD, exhibited association to percent predicted phenotypes.

**Conclusion:**

*GSTO2 *and *IL6R *are credible candidate genes for association to pulmonary function identified by GWA. These and other observed associations warrant replication studies. This resource of GWA results for pulmonary function measures is publicly available at http://www.ncbi.nlm.nih.gov/projects/gap/cgi-bin/study.cgi?id=phs000007.

## Background

Chronic obstructive pulmonary disease (COPD), which affects approximately six percent of the adult US population [1] and is the fourth most common cause of death in the US [2], has been defined as "airflow limitation that is not fully reversible" [3], a definition based on spirometry. Environmental factors, most notably tobacco smoking, are associated with accelerated longitudinal decline of pulmonary function and are important causes of COPD. Several lines of evidence indicate that genetic factors also contribute to the development of this condition. First, family studies have revealed increased risk of lung function impairment in smoking first degree relatives of COPD cases [4] and substantial heritability of spirometry measures in population based studies [5]. Second, severe alpha-1-antitrypsin deficiency due to homozygous mutations of the *SERPINA1 *(*AAT*) gene is a documented cause of COPD, although this condition explains only a small proportion of COPD in the population. Finally, family-based and case-control studies are beginning to reveal genetic variants other than those of the *SERPINA1 *gene that are associated with chronic airflow obstruction [6]. Despite this evidence of a genetic basis for susceptibility to COPD, the specific genetic risk factors underlying most cases of COPD remain uncertain.

The Framingham Heart Study offers the opportunity to conduct family-based linkage and association studies seeking potential genetic factors that influence obstructive (COPD, asthma), restrictive, or developmentally related lung function impairment. Using spirometry measurements as quantitative phenotypes, we have previously reported results of genome-wide linkage analyses among these same families using microsatellite markers spaced approximately 10 centiMorgans apart [7] and fine-mapping of a promising region on chromosome 6q [8,9]. The availability of data on over 100,000 single nucleotide polymorphisms (SNPs) throughout the genome now permits the application of genome-wide association (GWA) testing to the search for genetic risk factors for chronic airflow limitation. The longitudinal pulmonary function data that have been obtained over the years of the Framingham Heart Study, in combination with the 100K SNP data, make this a unique resource for the discovery of novel genetic risk factors for chronic airflow obstruction.

## Methods

Three types of spirometry phenotypes were evaluated for GWA: 1) measurements taken at a participant's most recent available examination and expressed as a percent of predicted; 2) the mean of measurements taken at two specified examinations; and 3) the annual rate of decline of spirometry measurements derived by calculating the slope of measurements across multiple examinations. All spirometry was performed without bronchodilator testing.

### Measurements as percent of predicted at latest examination

The spirometry measurements from each participant's latest examination with acceptable pulmonary function data [10] were used; eligible examinations included Cohort exams 19, 17, and 13 and Offspring exams 7, 6, 5, and 3. Predicted values for each lung function measurement were calculated using cohort and gender-specific regression models predicting spirometry measurements on the basis of age, age squared, and height squared [11] among Framingham subjects who were lifetime nonsmokers and had no history of chronic bronchitis, pulmonary disease, COPD/emphysema, asthma, or wheezing. The percent of predicted value was calculated by dividing the observed by the predicted value. Standardized residuals were then created by regressing the percent predicted on current smoking (y/n), former smoking (y/n), pack-years, and body mass index (BMI: kg/m^2^), in cohort and gender-specific models. Forced expiratory volume in one second (FEV_1_), forced vital capacity (FVC), forced expiratory flow between the 25^th ^and 75^th ^percentile (FEF_25–75_), the FEV_1_/FVC ratio, and the FEF_25–75_/FVC ratio were examined as cross-sectional percent of predicted measures. These phenotypes are referenced in tables preceded by the letters "pp" (for percent predicted), see Table 1 for explanation of phenotype abbreviations.

**Table 1:** Characteristics of phenotypes studied*

			Eligible exam cycles			
Phenotype	**N**^a^	Description	Offspring	Cohort	**Adjustment**^b^	Heritability
ppfev1	1217	Percent predicted FEV_1 _for latest exam	7, 6, 5, 3	19, 17, 13	Predicted defined by age, age^2^, height, % predicted adjusted for current or former smoking, pack-years, and BMI	0.36
ppfvc	1217	Percent predicted FVC for latest exam	7, 6, 5, 3	19, 17, 13	Same as ppfev1	0.45
ppratio	1217	Percent predicted FEV_1_/FVC for latest exam	7, 6, 5, 3	19, 17, 13	Same as ppfev1	0.29
ppfef	1212	Percent predicted FEF_25–75 _for latest exam	7, 6, 5, 3	19, 17, 13	Same as ppfev1	0.40
ppfefrat	1212	Percent predicted FEF_25–75_/FVC for latest exam	7, 6, 5, 3	19, 17, 13	Same as ppfev1	0.41
meanfev1	1222	Mean FEV_1 _from two exams	3 and 5	5 or 6 and 13	age, age^2^, BMI, height, current or former smoking, and pack-years	0.35
meanfvc	1222	Mean FVC from two exams	3 and 5	5 or 6 and 13	Same as meanfev1	0.51
meanratio	1222	Mean FEV_1_/FVC from two exams	3 and 5	5 or 6 and 13	Same as meanfev1	0.25
fev1slope	1097	Longitudinal slope of FEV_1_	7, 6, 5, 3	19, 17, 13	age, age^2^, height, height^2^, pack-years at first exam, interim pack-years, and sustained smoking	0.10
fefslope	1059	Longitudinal slope of FEF_25–75_	7, 6, 5, 3	19, 17, 13	Same as fev1slope	0.20

*Results for additional longitudinal slope phenotypes, residual from predicted phenotypes, phenotypes created from spirometry at a single exam, and phenotypes limited to smokers reporting 10 or more pack-years are available at http://www.ncbi.nlm.nih.gov/projects/gap/cgi-bin/study.cgi?id=phs000007

a) sample size with phenotype out of 1345 with GWA genotyping

b) adjustment performed in four separate regression models by sex and Original or Offspring cohort

### Mean of measurements at two specified examinations

In previous analyses of the genetics of lung function in the Framingham families [7,8], we have used the mean of the values of each spirometry measure from two specified examinations. For Cohort participants, spirometry data from exam cycles 5 or 6 and cycle 13 were used to generate the mean value. In Offspring participants, spirometry data from cycle 3 and cycle 5 were used to generate the mean value. The mean FEV_1_, FVC, and FEV_1_/FVC ratio were adjusted for the effects of age, age^2^, BMI, height, dummy variables indicating never, former, or current smoking status, and, for former and current smokers, pack-years. Standardized residuals were generated separately by sex and within Cohort or Offspring samples. These phenotypes are referenced in tables as meanfev1, meanfvc, and meanratio.

### Annual rate of decline of measurements

Rate of decline phenotypes were defined by fitting a slope to the spirometry data from different time points. The examinations incorporated were the same as those eligible for percent of predicted phenotypes described above. Slopes were calculated by ordinary least-squares using all available data. A minimum of two eligible exams were needed to calculate a slope. Slopes were adjusted for the covariates age, age squared, height, and height squared, using mean ages and heights from included exams. Slopes were also adjusted for pack-years at first exam, interim pack-years, and sustained smoking (y/n). FEV_1 _and FEF_25–75_, the slope phenotypes with the highest heritability, were studied in the GWA. These phenotypes are referenced in tables as fev1slope and fefslope.

### Statistical analysis methods

All SNPs were studied using family-based association tests (FBAT) and generalized estimating equations (GEE) [12] (see 100K Overview). SNP results reported met the criteria of having a minor allele frequency ≥10%, a Hardy-Weinberg p-value ≥ 0.001, and a call rate ≥ 80%. All reported FBAT tests also required a minimum of ten informative families.

Multipoint variance component linkage analysis was implemented with a subset of 10,588 SNPs and all 612 available microsatellites studied in previous linkage analyses [12]. Multipoint identity-by-descent estimates were generated using the software Merlin [13]. Heritability estimates, estimating the proportion of the total phenotypic variance due to genetic effects, and variance component linkage analysis were performed using the software SOLAR [14].

In addition to evaluating all SNP associations with each phenotype individually, we developed a method to identify SNPs in or near genes that exhibited the strongest associations (as assessed by p-value) to multiple spirometry phenotypes. For each phenotype, we identified the 200 lowest p-values that met the criteria above and were localized within 60,000 base pairs of the transcription start or stop of a gene. All gene annotations are derived from the UCSC genome browser May 2004 assembly, build 125 http://genome.ucsc.edu/[15,16]. We evaluated the frequency that a SNP appeared among the 200 lowest p-values in gene regions for the ten phenotypes. This strategy was based on the hypothesis that SNPs identified to be associated with multiple spirometry measures are more likely to reflect a true association with lung function than SNPs identified to be associated with only a single measurement. SNPs in gene regions that appeared among the lowest 200 p-values in five or more of the phenotypes studied are reported.

### Candidate genes

Genes previously reported in the literature to be associated with spirometry measures or pulmonary disease were examined to determine whether any available 100K SNPs in or near the genes were associated with spirometry phenotypes. Twelve COPD candidate genes studied in the Boston Early-Onset COPD cohort [17], and the *SERPINE2 *gene, a novel gene identified through linkage and association with COPD in the same cohort [6], were reviewed. The previously established COPD gene alpha-1-antitrypsin (*SERPINA1*) and the cystic fibrosis transmembrane conductance regulator (*CFTR*) as well as additional genes in the class of Glutathione S-transferases (O1, O2, M2, T1, T2) and surfactant proteins (*SFTPA1*, *SFTPC*) were reviewed. In addition, extracellular super oxide dismutase (*SOD3*) [18,19], interleukin-8 receptor alpha (*IL8RA*) [20], interleukin-10 (*IL10*) [21], beta-2 adrenergic receptor (*ADRB2*) [22], and transforming growth factor beta-1 (*TGFB1*) [23] were examined as COPD candidates. The GEE and FBAT SNP association results in or within 60 kilobase pairs (kb) of these 27 genes was reviewed. By specifying a 60 kb distance around the gene to screen results, we were able to identify SNPs near most candidate gene regions, but often the SNP reported does not lie strictly within the transcription start or stop of the gene of interest.

## Results

Table 1 reports the heritability estimates for each of the ten phenotypes presented. The slope phenotypes have lower heritability than their corresponding cross sectional phenotypes. FVC has the highest heritability among phenotypes defined using the same method (percent predicted or mean). The percent predicted FEF_25–75_/FVC ratio had a higher heritability estimate than either FEV_1_/FVC ratio phenotype.

Table 2 reports the SNPs with the lowest p-values for any of the 10 phenotypes evaluated in GWA analysis. Results were ranked by p-value and the top 25 SNPs are reported. In some cases, a SNP had top ranked results for multiple correlated phenotypes, and thus the p-value for all phenotypes is reported at the ranking position of the best p-value. Table 2a was ranked by GEE p-value, and Table 2b was ranked by FBAT p-value. SNP positions are reported according to NCBI Human Genome Build 35. SNPs in the Affymetrix set whose chromosome and position are unknown and SNPs on sex chromosomes are not reported for association. In total, 70,987 SNPs were considered in association analyses. None of these results achieved a conservative threshold for genome-wide significance. All results, regardless of allele frequency, call rate, or deviation from Hardy-Weinberg are publicly available at http://www.ncbi.nlm.nih.gov/projects/gap/cgi-bin/study.cgi?id=phs000007.

**Table 2:** GEE, FBAT, and linkage results

2a Top Association results based on GEE p-value						
**Phenotype**	**SNP**	**Chr**	**Physical position**	**GEE p-value**	**FBAT p-value**	**Gene Region**

fev1slope	1. rs3867498	15	22629880	**1.36 × 10^-06^**	0.07	*SNRPN*
meanfvc	2. rs441051	7	93698651	**2.16 × 10^-06^**	0.005	*COL1A2*
meanratio	3. rs2838815	21	45454018	**2.63 × 10^-06^**	0.0002	*ADARB1*
ppfvc	4. rs1455782	15	90851970	**4.23 × 10^-06^**	0.18	*FLJ32831*
meanfvc	5. rs10516541	4	108472826	**4.32 × 10^-06^**	7.19 × 10^-05^	
meanfev1				**1.7 × 10^-05^**	0.0002	
ppratio	6. rs310558	8	51575144	**5.14 × 10^-06^**	0.14	*SNTG1*
ppfev1	7. rs3820928	2	227598971	**5.33 × 10^-06^**	0.0005	*RHBDD1*
ppfef	8. rs730532	14	51588561	**5.89 × 10^-06^**	0.15	*NID2*
ppfefrat	9. rs808225	14	57467669	**7.38 × 10^-06^**	0.0008	
ppratio				**1.45 × 10^-05^**	0.007	
ppfef	10. rs4129267	1	151239337	**7.39 × 10^-06^**	0.07	*IL6R*
ppfev1	11. rs2906966*	17	15272242	**8.31 × 10^-06^**	5.03 × 10^-05^	*CDRT4*
ppfvc	12. rs357394	7	137506642	**8.77 × 10^-06^**	0.14	
fefslope	13. rs1994169	12	33436783	**9.55 × 10^-06^**	0.05	*SYT10*
meanratio	14. rs2225434	21	45458574	**9.68 × 10^-06^**	0.0004	*ADARB1*
meanfvc	15. rs156697^(a)^	10	106029175	**9.78 × 10^-06^**	9.42 × 10^-05 ^	*GSTO2*
meanfev1				**1.8 × 10^-05^**	0.002	
ppfefrat	16. rs564425	13	46797096	**1.03 × 10^-05^**	0.02	
ppfef	17. rs10498441	14	51613974	**1.06 × 10^-05^**	0.28	*NID2*
ppratio	18. rs880713	2	128847844	**1.11 × 10^-05^**	0.26	*AK128224*
ppfefrat	19. rs811732	14	57456738	**1.3 × 10^-05^**	0.20	
ppfvc	20. rs6558132	8	29526866	**1.58 × 10^-05^**	0.74	
fev1slope	21. rs6972823	7	3009137	**1.69 × 10^-05^**	0.10	
ppfef	22. rs9285611^(b)^	1	81895076	**1.7 × 10^-05^**	0.07	
meanfev1	23. rs10504836	8	88669709	**1.72 × 10^-05^**	0.28	
meanfev1	24. rs1491520	3	193243153	**1.77 × 10^-05^**	0.12	
ppfvc	25. rs9300826	13	103001176	**1.79 × 10^-05^**	0.11	

**2b Top Association results based on FBAT p-value**						

**Phenotype**	**SNP**	**Chr**	**Physical position**	**GEE p-value**	**FBAT p-value**	**Gene Region**

ppratio	1. rs10922530	1	89139273	0.001	**8.7 × 10^-07^**	*CCBL2*
ppfefrat				0.002	**3.78 × 10^-06^**	
ppfef				0.005	**2.36 × 10^-05^**	
ppfefrat	2. rs3753683	1	89139592	0.004	**1.87 × 10^-06^**	*CCBL2*
ppratio				0.004	**3.59 × 10^-06^**	
ppfef				0.01	**1.42 × 10^-05^**	
ppratio	3. rs219349	14	59525168	0.01	**6.15 × 10^-06^**	*LRRC9*
ppfefrat				0.04	**4.91 × 10^-05^**	
ppratio	4. rs219391	14	59554684	0.02	**6.18 × 10^-06^**	*LRRC9*
ppratio	5. rs219326	14	59512943	0.02	**8.51 × 10^-06^**	*LRRC9*
ppfefrat				0.06	**3.74 × 10^-05^**	
ppfefrat	6. rs1409149	1	89141170	0.005	**9.12 × 10^-06^**	*CCBL2*
ppratio				0.005	**1.08 × 10^-05^**	
ppfef				0.01	**5.14 × 10^-05^**	
ppfvc	7. rs9299191	9	110944100	0.009	**1.79 × 10^-05^**	
meanfvc	8. rs10515289^(c)^	5	99315845	0.17	**2.21 × 10^-05^**	
ppfvc	9. rs10498137	2	222966943	0.05	**2.23 × 10^-05^**	*PAX3*
ppfef	10. rs3858282	10	90424391	0.001	**2.92 × 10^-05^**	*LIPF*
ppratio	11. rs905367	4	59896363	0.02	**2.95 × 10^-05^**	
meanfvc	12. rs10495872	2	37915266	0.05	**3.02 × 10^-05^**	
fev1slope	13. rs1347222	12	80896622	0.01	**3.27 × 10^-05^**	
ppratio	14. rs2009488	4	59895895	0.03	**3.43 × 10^-05^**	
meanfvc	15. rs6481257	10	58608558	0.02	**3.52 × 10^-05^**	
ppratio	16. rs461951	14	59608986	0.02	**3.63 × 10^-05^**	*LRRC9*
meanfev1	17. rs491552	6	151403169	0.01	**3.86 × 10^-05^**	*MTHFD1L*
meanfev1	18. rs1910137	4	27087329	0.08	**4.04 × 10^-05^**	
ppfvc	19. rs2831605^(d)^	21	28467064	0.03	**4.41 × 10^-05^**	
ppfev1	20. rs2906966*	17	15272242	8.31 × 10^-06^	**5.03 × 10^-05^**	*CDRT4*
fefslope	21. rs6740919	2	67531706	0.0009	**5.17 × 10^-05^**	*ETAA16*
meanfev1	22. rs10498818	6	63134129	0.02	**5.25 × 10^-05^**	
ppfefrat	23. rs1393593	4	59907689	0.06	**5.44 × 10^-05^**	
ppfefrat	24. rs9312080	4	59891451	0.11	**5.63 × 10^-05^**	
ppfefrat	25. rs7851363	9	20748306	0.006	**5.7 × 10^-05^**	*KIAA1797*

**2c Linkage peaks with LOD score > 2.0**						

**Phenotype**	**SNP**	**Chr**	**Physical position**	**1.5-LOD interval start**	**1.5-LOD interval end**	**LOD score**

meanfev1	rs2300081	6	168097526	165513097	170788550	2.89
fefslope	rs5909594	X^(e)^	118057198	113970434	124311695	2.86
ppfev1	AGC001b	6	170788550	164268152	170788550	2.65
ppratio	rs10497042	2	150675813	148296793	154203261	2.40
meanratio	rs10518669	1	82811739	74999965	88728520^(b)^	2.37
ppfef	rs2974490	5	113433775	95608129	124687730^(c)^	2.29
meanfvc	rs10488908	21	21904704	19805361	34461762^(d)^	2.29
ppfev1	rs10518032	4	170322764	161768472	181501690	2.28
ppfvc	rs10489542	1	225169260	213625196	233478438	2.17
ppfef	rs721411	17	36902470	28313357	52519635	2.17
ppfev1	rs753765	17	57323782	45345729	59677087	2.17
meanfev1	rs4918762	10	114389096	102080914	122214789^(a)^	2.12
ppfvc	rs10517825	4	166251091	156507719	191091333	2.02

*) SNP occurring in both Table 2a and 2b

a) SNP associated to meanfvc and meanfev1 in Table 2a under linkage peak for meanfev1 on chromosome 10.

b) SNP associated to ppfef in Table 2a under linkage peak for meanratio on chromosome 1.

c) SNP associated to meanfvc in Table 2b under linkage peak for ppfef on chromosome 5.

d) SNP associated to ppfvc in Table 2b under linkage peak for meanfvc on chromosome 21.

e) X chromosome linkage results are not available online

Only a single SNP among those reported in Tables 2a and 2b is a known coding SNP. Among the top ranked GEE p-values was a non-synonymous coding SNP (rs156697) in the Glutathione S-transferase omega 2 gene (*GSTO2*) on chromosome 10. The SNP was among the top ranked GEE p-values for association with the mean FEV_1 _and mean FVC phenotypes.

Other SNPs reported in Table 2a were localized to intronic gene regions in *COL1A2*, *ADARB1*, *SNTG1*, *RHBDD1*, *NID2*, *IL6R*, and *SYT10*. The best GEE p-value localized to the untranslated region of *SNRPN*. SNPs identified by FBAT p-value, reported in Table 2b, located in introns were in *CCBL2*, *LRRC9*, *PAX3*, *LIPF*, *MTHFD1L*, and *KIAA1797*. Additional genes reported in Tables 2a and 2b were within 60 kb of the associated SNP. SNP rs2906966, among the top 25 p-values for both tests, is located near *CDRT4*. SNPs reported may be in linkage disequilibrium (LD). Among the top six FBAT results, strong LD was observed between the three chromosome 1 SNPs in *CCBL2*, and the three chromosome 14 SNPs in *LRRC9*, thus only 2 regions are being identified.

Linkage to all autosomes and the X chromosome was performed. Table 2c reports all LOD scores above 2.0 with the 1.5-LOD support interval. The best LOD score observed is in a region of linkage on chromosome 6q that was reported previously using microsatellites in the Framingham families [7,8]. The original LOD score of 2.4 for mean FEV_1 _using genome-wide microsatellites [7] was increased to a LOD of 2.89 with the addition of SNP data, and a LOD of 2.65 was observed in the same region for the percent predicted FEV_1 _phenotype. The second highest LOD score observed was 2.86 for the longitudinal FEF_25–75 _phenotype, which was located on the X chromosome. The percent predicted FEV_1 _and FVC phenotypes both had LOD scores over 2.0 on chromosome 4, with overlapping LOD support intervals centered around 166–170 Mb.

Table 3 reports SNPs that met the quality control criteria and were among the top 200 p-values for SNPs located within 60 kb of a gene for at least five of the phenotypes studied. A SNP in an intron of the interleukin 6 receptor (*IL6R*) and a SNP near the sodium channel, voltage gated, type I alpha (*SCN1A*) gene were among the top ranked result for six phenotypes. All others were among the top ranked results for five phenotypes. The genes syntrophin, gamma 1 (*SNTG1*) and the chromosome 20 open reading frame 133 (*C20orf133*) appear in both the FBAT and GEE lists of top results across 5 phenotypes. The two SNPs in *SNTG1 *are separated by 104 kb and have low LD (r^2 ^= 0.05 and D' = 0.28 in the HapMap CEU sample). The two *C20orf133 *SNPs are in strong LD (r^2 ^= 0.78 in HapMap CEU), though rs10485771 is located 3' of the gene.

**Table 3:** Top ranked SNPs within 60 kb of a gene and associated with five or more of the phenotypes studied

SNP	Chr	bp position	Gene region
**3a GEE**						

rs4129267	1	151239337	*IL6R*
rs7587026	2	166804257	*SCN1A*
rs3820928	2	227598971	*RHBDD1*
rs445347	5	53011487	*NDUFS4*
rs310558	8	51575144	*SNTG1*
rs581446	18	10646848	*FAM38B*
rs10485770	20	13943214	*C20orf133*

**3b FBAT**						

rs10489030	4	24521105	3' of *SOD3*
rs2438345	5	90198041	*GPR98*
rs7759033	6	116404207	*FRK*
rs2391996	7	31487207	*C7orf16*
rs10504106	8	51471079	*SNTG1*
rs10485771	20	13999103	*C20orf133*

Table 4 reports the best p-value observed when examining SNP results specifically in the regions of the 27 candidate genes. SNPs reported are within 60 kb of the transcription start or stop for the gene listed, and sometimes lie within a different gene. Only 20 of the 27 genes of interest had SNPs in or within 60 kb of the gene. No SNPs were near enough to the genes *GSTM1 *and *TNF*, presented by Hersh et al. (2005), and *GSTO1*, *GSTT1*, *SFTPA1*, *SFTPC *and *IL8RA *to be considered. Of the 20 best FBAT results, 12 were p-values less than 0.05, but only 5 were p-values less than 0.01. Of the best GEE results, 14 were p-values less than 0.05, and 5 were p-values less than 0.01. Three gene regions had p-values less than 0.01 for both types of test, and these were *CFTR*, *GSTO2*, and *SOD3*. The vitamin D binding protein (*GC*) region produced an FBAT p-value of 0.0009, though GEE results were weaker.

**Table 4:** Candidate gene evaluation

Gene region	Chr	SNP	Phenotype	FBAT p-value	GEE p-value
Alpha-1-antitrypsin (*AAT/SERPINA1*)	14	rs2402446 rs10484042	ppfvc fev1slope	0.02 0.90	0.30 0.01
Beta-2 adrenergic receptor (*ADRB2*)	5	rs30329 rs9325117	fefslope meanratio	**0.009** -	0.07 0.04
cystic fibrosis transmembrane regulator (*CFTR*)	7	rs213987 rs10487367	meanfvc ppratio	**0.006** 0.25	0.38 **0.002**
Microsomal epoxide hydrolase (*EPHX1*)	1	rs3738051	fefslope ppfefrat	0.01 0.03	0.30 0.04
Vitamin D binding protein (*GC*)	4	rs423817 rs842873	ppfefrat ppfefrat	**0.0009** 0.05	0.06 0.02
Glutathione S-transferase M2 (*GSTM2*)	1	rs542338	meanratio fev1slope	0.19 0.43	0.63 0.20
Glutathione S-transferase O2 (*GSTO2*)	10	rs156697	meanfvc	**9.4 × 10^-05^**	**9.8 × 10^-06^**
Glutathione S-transferase P1 (*GSTP1*)	11	rs688878	ppfefrat ppfvc	0.07 0.39	0.35 0.02
Glutathione S-transferase T2 (*GSTT2*)	22	rs140289	ppfvc ppfev1	0.17 0.41	0.13 0.10
Heme oxygenase (*HMOX1*)	22	rs2267331 rs10483190	fev1slope meanratio	0.07 0.82	0.80 **0.008**
Interleukin-10 (*IL10*)	1	rs10494879	fefslope fev1slope	0.06 0.46	0.73 0.02
Matrix metalloproteinase-1 (*MMP1*)	11	rs495366	fefslope ppfef	0.02 0.20	0.13 0.03
Matrix metalloproteinase-9 (*MMP9*)	20	rs2903908	ppfefrat	0.19	0.05
Alpha-1-antichymotrypsin (*SERPINA3*)	14	rs10484047	ppfvc fev1slope	0.02 0.10	0.03 0.01
Serine proteinase inhibitor E2 (*SERPINE2*)	2	rs717610	meanfvc meanratio	0.04 0.19	**0.003** **0.001**
Surfactant protein B (*SFTPB*)	2	rs7577293	meanratio	0.02	0.06
Surfactant protein D (*SFTPD*)	10	rs726289	fefslope	0.02	0.03
extracellular super oxide dismutase (*SOD3*)	4	rs10489030	ppfev1 ppfvc	**0.0005** 0.01	0.02 **0.007**
transforming growth factor beta-1 (*TGFB1*)	19	rs3745295	fev1slope ppfev1	0.21 0.74	0.62 0.18
Tissue inhibitor of metalloproteases-2 (*TIMP2*)	17	rs2889529	ppfef meanratio	0.07 0.18	0.11 0.09

Best p-value for FBAT and GEE tests reported with specific SNP*phenotype producing result. P-values < 0.01 bolded.

## Discussion

This is the first GWA of quantitative lung function measures to be reported, and it provides an opportunity for both hypothesis generation and hypothesis testing. We have identified a number of novel gene regions associated with pulmonary function. Associations with these SNPs and gene regions require replication in other study samples as well as functional studies before any statement about causality is warranted. Many of the best p-values are likely to reflect false positive results, and GEE results exhibited elevated Type I error [12] (see 100K Overview). Additional studies of this data will be useful, including smoking stratified analyses and more sophisticated approaches to creating multivariate phenotypes. However, several of the observed associations involve genes for which there are plausible biologic rationales for a relation to lung function phenotypes.

The Glutathione S-Transferase (GST) superfamily genes are of interest because of their role in metabolism of xenobiotics, such as cigarette smoke. Recently, Hersh et al. studied *GSTP1 *and *GSTM1 *in two independent analyses of COPD and reported null findings. In contrast, a study of annual change in lung function measures in a population based cohort reported that the *GSTT1 *deletion alone or in combination with the *GSTM1 *deletion influenced decline in FEV_1 _in men [24]. Using the Affymetrix 100K SNP GeneChip, we have limited ability to directly confirm or refute the aforementioned findings because no SNPs were genotyped within 10 kb of the genes.

Here, we show that a non-synonymous SNP in exon 5 of *GSTO2 *encoding an Asn142Asp amino acid change is among the most striking GWA results for mean FEV_1 _and FVC phenotypes. Both the non-synonymous SNP, rs156697, and a second SNP, rs156699, located in an intron exhibited strong association using both FBAT and GEE tests (r^2 ^between SNPs = 0.8 in HapMap CEU). Linkage results also support the evidence for *GSTO2*, as the gene's position lies within the confidence interval around the LOD of 2.12 observed on chromosome 10 for mean FEV_1 _(Table 2c). *GSTO2 *is involved in the biotransformation of arsenic, which is a component of cigarette smoke, and may exhibit modest expression in bronchial epithelial cells. Gene expression studies in COS-1 cells demonstrated that the Asp142 variant exhibited 76% of the level of expressed protein occurring in the wild-type, and expression levels were further reduced to 15% when the Asp142 occurred in conjunction with an Ile158 variant [25]. The observation of strong association to a non-synonymous polymorphism with demonstrated effects on gene expression is compelling. Moreover, this finding in conjunction with a growing literature on GST gene association with pulmonary phenotypes suggests that a complete evaluation of functional variants in this gene family may be warranted.

The *IL6R *SNP was not only among the top 25 p-values for percent predicted FEF_25–75_, but also among the top 200 p-values in gene regions for six of the ten phenotypes presented. *IL6R *is thought to be expressed in lung, and may play a role in the immune response. Recently, we have shown that IL6 levels in blood were associated with impaired lung function in the Framingham offspring cohort [26]. The IL6 pathway, as a mediator of the inflammatory process, is of interest as it relates to lung function phenotypes.

The SNP identified in the *SOD3 *region lies within a hypothetical protein 3' of the *SOD3 *gene. The non-synonymous SNP in *SOD3 *that has been reported for association with COPD (rs1799895) was not included in the HapMap, so we could not determine the extent of LD between it and the SNPs genotyped in this study. Another exon 3 SNP (rs2536512) located 519 base pairs away from rs1799895 is present in the HapMap. The associated SNP identified in this study, rs10489030, is in very low LD with the *SOD3 *exon 3 HapMap SNP (D' = 0.32 and r^2 ^= 0.005 in HapMap CEU). The low LD between the *SOD3 *exonic SNP and this 3' SNP, separated by 43.5 kb, does not suggest a clear replication of association with the *SOD3 *gene, but suggests that the genomic region is of continued interest. Also on chromosome 4, a SNP in the region of the vitamin D binding protein (*GC*) was associated with the percent predicted FEF_25–75_/FVC ratio with a p-value of 0.0009 using the FBAT test, but this SNP is in low LD with the Asp432Gly polymorphism, rs7041 (D' = 0.17 and r^2 ^= 0.007 in HapMap CEU).

The SNP identified in the region of *SERPINE2 *(rs717610) was not in LD with the six reported SNPs replicating significant associations to COPD [6] that were also available in HapMap, as the r^2 ^values ranged from 0.002 to 0.008. Two of the top SNPs (rs3820928, GEE rank #7; rs10498137, FBAT rank #9) lie within the linkage region identified for FEV_1_/FVC in severe early-onset COPD cases [27] that subsequently led to the discovery of the *SERPINE2 *associated SNP. SNP rs3820928 was among those identified with association to five of the phenotypes studied and lies in the gene *RHBDD1 *(alias *DKFZp547E052*). Not much is known about the function of this gene, but the associated SNP is located in a region with LD extending to the adjacent *COL4A4 *gene. The rs3820928 exhibited an r^2 ^of 0.81 with two non-synonymous coding SNPs in *COL4A4 *in the HapMap CEU data. Defects in Type IV collagen genes have been shown to influence Goodpasture's syndrome, an autoimmune disease affecting the lung [28], and both *COL4A4 *and *COL4A3 *lie in this region, sharing a common promoter. These results do not provide a strong replication of the original *SERPINE2 *SNP associations due to the low LD between SNPs reported in the literature and the SNPs from the 100K data with association. However, the results suggest that chromosome 2q is of continued interest and may harbor multiple genes influencing lung function.

Studying lung function measures in a community-based sample may identify genetic variants associated with lung growth and development, susceptibility to obstructive ventilatory impairment related to asthma, emphysema and COPD or susceptibility to restrictive ventilatory impairment due to pulmonary fibrosis or other processes. The relevance to disease pathogenesis of associations between SNPs and lung function must be interpreted with caution, and some of the observed associations may reflect polymorphisms that protect against ventilatory impairment by leading to better lung function in early life or protection against the adverse effects of cigarette smoking. All of the GWA results are publicly available at http://www.ncbi.nlm.nih.gov/projects/gap/cgi-bin/study.cgi?id=phs000007. Replication of novel results identified by GWA will be the true test of the value of the GWA approach to gene discovery.

## Conclusion

These publicly available results provide a resource for investigators to assess whether their findings of association to pulmonary function phenotypes replicate in the Framingham population. In addition, we have identified novel results that warrant replication studies in other populations.

## Abbreviations

BMI = body mass index; COPD = chronic obstructive pulmonary disease; FEV_1 _= forced expiratory volume in one second; FBAT = family based association test; FVC = forced vital capacity; FEF_25–75 _= forced expiratory flow between the 25^th ^and 75^th ^percentile; GEE = generalized estimating equation; GWA = genome-wide association; kb = kilobase pairs (1000 base pairs); LD = linkage disequilibrium; SNPs = single nucleotide polymorphisms.

## Competing interests

The authors declare that they have no competing interests.

## Authors' contributions

JBW created the phenotypic residuals that were analyzed, reviewed GWA results, and drafted the manuscript text and tables. REW, DJG, and GTO assessed the quality of spirometry data, identified exclusion criteria, and defined the covariates and methods to develop phenotypes for analysis. JML wrote scripts to parse through the GWA results and facilitate the presentation of results. All authors read and approved the final manuscript.
